# The Presence of Bacteriophages in the Human Body: Good, Bad or Neutral?

**DOI:** 10.3390/microorganisms8122012

**Published:** 2020-12-16

**Authors:** Marzanna Łusiak-Szelachowska, Beata Weber-Dąbrowska, Maciej Żaczek, Jan Borysowski, Andrzej Górski

**Affiliations:** 1 Bacteriophage Laboratory, Ludwik Hirszfeld Institute of Immunology and Experimental Therapy, Polish Academy of Sciences, 53-114 Wrocław, Poland; marzanna.lusiak-szelachowska@hirszfeld.pl (M.Ł.-S.); beata.weber-dabrowska@hirszfeld.pl (B.W.-D.); maciej.zaczek@hirszfeld.pl (M.Ż.); 2 Phage Therapy Unit, Ludwik Hirszfeld Institute of Immunology and Experimental Therapy, Polish Academy of Sciences, 53-114 Wrocław, Poland; 3 Department of Clinical Immunology, Transplantation Institute, Medical University of Warsaw, 02-006 Warsaw, Poland; jborysowski@interia.pl; 4 Infant Jesus Hospital, Medical University of Warsaw, 02-005 Warsaw, Poland

**Keywords:** bacteriophages, human health, human microbiome, fecal microbiota transplantation, phage therapy

## Abstract

The presence of bacteriophages (phages) in the human body may impact bacterial microbiota and modulate immunity. The role of phages in human microbiome studies and diseases is poorly understood. However, the correlation between a greater abundance of phages in the gut in ulcerative colitis and diabetes has been suggested. Furthermore, most phages found at different sites in the human body are temperate, so their therapeutic effects and their potential beneficial effects remain unclear. Hence, far, no correlation has been observed between the presence of widespread crAssphage in the human population and human health and diseases. Here, we emphasize the beneficial effects of phage transfer in fecal microbiota transplantation (FMT) in *Clostridioides difficile* infection. The safety of phage use in gastrointestinal disorders has been demonstrated in clinical studies. The significance of phages in the FMT as well as in gastrointestinal disorders remains to be established. An explanation of the multifaceted role of endogenous phages for the development of phage therapy is required.

## 1. Introduction

Bacteriophages (phages) are bacterial viruses that are the most abundant, omnipresent, and diversified biological group inhabiting Earth [1,2]. They are detected in soil, water and in the human body (feces, saliva, sputum, blood, and urine) [3]. Phages can be found from blood and other tissues after administration through different routes [4]. Interestingly, phages may pass the intestinal barrier and get translocated to blood, lymph and internal organs [5]. Intestinal epithelial cells (IECs) play an essential role in the absorption of nutrients and constitute a barrier against potentially harmful microbial antigens [6]. The in vivo role of epithelial cells in the uptake of lumen antigens such as viruses, bacterial cell debris and dietary particles was investigated in mouse experiments. Goblet cell-associated passageway (GAP) enables the entry of soluble protein antigens, but not inert particles (0.02–2 µm) into lamina propria (LP), the tissue that lies under the epithelium [7]. Small molecular weight antigens such as chicken ovalbumin, dextran, and bacterial LPS enter the LP via GAP [6]. However, epithelial cells overlying the villi can internalize antigens such as bacterial cell debris and inert nanoparticles, which are then found colocalizing with the CD11c+ dendritic cells in the LP. 20–40 nm nanoparticles are taken up readily by IECs, while nanoparticles larger than 100 nm are taken up mainly by the epithelial cells overlying Peyer’s patches [6].

The intestines are predominantly inhabited by phages and, to a lesser degree, eukaryotic viruses [8]. One could expect as estimated that 10^15^ phages reside in the human gut, which accounts for approximately 10^8^–10^10^ phages per gram of human stool depending on the extraction method used [9,10,11,12] and ~10^9^ bacterial cells per gram of stool [13]. As estimated by month one of infants life virus-like particles (VLPs) counts averaged 1.6 × 10^9^ per gram of stool, at month 4 were similar [14]. In 2-to 5-year-old children, VLPs counts averaged 9.4 × 10^8^ per gram of stool. This is similar to that estimated for adults. For months one and four, most samples were positive for bacterial 16SrRNA gene sequences with a median value of 3.1 × 10^8^ [14]. Individual specificity and temporal stability of the human gut virome were observed for up to 1 year [15]. What is more, phage content in human stool samples may vary depending on the health status of the donor in some gastrointestinal disorders. In Łusiak-Szelachowska et al.’s (2008) analysis, the number of coliphages was greater in patients with disorders predisposed to cancer development and those with colorectal cancer than in healthy subjects [16]. Moreover, the metagenomic approach showed that the majority of found phage sequences are not yet identified; for instance, RNA phage [9]. Moreover, the gut bacteria harbor a large number of prophages whose biological role is unclear. Phages can potentially alter gut bacterial microbiota. It has been suggested that changes in phage composition may play a role in some diseases [17,18]. Phage may also play an important role in microbiota homeostasis. They may regulate microbiota diversity and may be involved in an increase in gut permeability and immune disturbances [19]. Sutton and Hill (2019) pointed out the critical need for extensive optimization and validation of all steps of virome analysis protocols to bioinformatic pipelines as well as the extensive study of the role of phages in shaping the human gut microbiome in the aspects of possible phage use as a therapeutic tool [20].

## 2. Phages in Healthy Individuals

Intestinal microbiota consists of bacteria, viruses (including phages) and fungi. The small intestine contains such bacteria as *E. coli*, *Streptococcus* spp. and *Bacteroidetes*. The large intestine and stool samples include the dominant bacteria *Firmicutes* and *Bacteroidetes* [21,22]. Phages that are detected in the intestine belong to the order *Caudovirales* with double-stranded DNA (families *Siphoviridae, Myoviridae* and *Podoviridae*) as well as the other phages with single-stranded DNA (families *Microviridae* and *Inoviridae*) [21,22]. The prevalence of crAssphage infecting *Bacteroidetes* in fecal samples in half of the healthy individuals from 4 cities in two continents was observed [23]. Most phages in different places in the human body are temperate. The fecal samples from healthy individuals contained mainly temperate phages (56.5%) vs. virulent phages (2.5%), whereas patients with digestive or respiratory system diseases and leukemic disease also had temperate phages (54.8–56%) vs. higher frequency of virulent phages (13–20%) compared with healthy individuals [24]. Lower titers of temperate phages were found in fecal samples collected from healthy individuals and from patients, whereas higher titers of virulent phages were detected from patients. The temperate phages belonged mainly to λ type and φ80 type [24]. A study of HLA-matched infants up to 3 years of age was performed. In 10 cases with autoantibodies (6 with no progression to type 1 diabetes (T1D) and 4 who developed (T1D) and 8 cases non-seroconverted control individuals), most intestinal *E. coli* phages were temperate (63 phages) and only in a few samples were lytic *Enterobacteria* phages found [25]. However, a recent study indicated that the gut virome of 9 out of 10 healthy human subjects had no dominance of phage sequences harboring marker genes of temperate phages (integrase and site-specific recombinase genes) [15]. These results led the authors to propose that mechanisms other than the integration of the phage genome into the host may be responsible for the long-term persistence of phages in the human gut. Sutton and Hill (2019) discussed actual data regarding the human virome and the influence of phages on gut microbiome shape [20]. According to current knowledge, phages are the most predominant element of the human microbiome, and they are believed to play a crucial role in shaping microbial composition driving bacterial diversity and facilitating horizontal gene transfer. Double-stranded DNA phages of the order *Caudovirales* are one of the dominant fractions of the gut virome. The concept of a core human virome was based on the fact that 23 core phages (based on the presence of phage contigs from the healthy individuals) were observed in more than 50% of samples from an independent group of 62 healthy individuals [26]. In a differing opinion, the human gut microbiome is highly individual-specific at a sequence level [20]. Additionally, it was reported that phages that colonize the gut are unique to each individual and with a high degree of interpersonal variation [27]. Intestinal mucosa plays an essential role in maintaining human health. The quantitative and qualitative differences are between phages of healthy individuals and patients [27,28]. Zuo et al. (2019) indicated a lower abundance of *Caudovirales* phages in the gut mucosa of healthy individuals, but a higher richness and diversity in these phages were observed in comparison to ulcerative colitis (UC) patients [28]. Manrique et al. (2017) suggest that an adequate balance between lysis and lysogeny in the human gut is essential to maintain human health [29]. In a healthy human gut, only a small part of the prophage reservoir is activated and found as extracellular phages. The model of the shift from health to disease has been proposed, which was based on the increase of some prophage induction, which is likely sensitive to stress. Therefore, the process, such as inflammation in patients with inflammatory bowel disease (IBD), may lead to an increase in prophage activity [29]. The increase in lytic phages in stool has also been correlated with the digestive system and leukemic diseases [24]. Another study emphasizes that phage particles may harbor antibiotic resistance genes (ARGs) in healthy individuals [30]. One hundred fifty fecal samples from healthy individuals who did not receive antibiotic treatment in the 3 months prior to sample collection were examined. In 72.7% of stools of healthy individuals, at least one ARG in phage DNA was found.

## 3. Phages in Diseases

Recent reports have demonstrated that human gut microbiota may be altered in various disorders such as obesity, diabetes, metabolic disorders, diarrhea, IBD, and malnutrition [17,21,31]. Garmaeva et al. (2019) reported that it is difficult to determine whether changes in the virome and microbiome are a cause or an effect of the disease. Koch’s criteria refer to the causal relationship between microbe and disease. The metagenomic version of Koch’s postulates relates to significant differences in metagenomic traits between sick and healthy individuals and evidence that inoculation of samples from sick to healthy animals leads to the induction of the disease. Only some criteria are supported in human phage research, e.g., significant differences in viral contigs or specific phages between sick and healthy individuals, and only a few animal studies have been performed and mostly with fecal microbiota transplantation (FMT) application rather than inoculation of phages [32].

Recent studies have indicated an increase of gut permeability in mammals (a rat model) after exposure to commercial phage cocktails (lysate) against *Enterobacteriaceae, Staphylococcaceae, Streptococcaceae* and *Pseudomonadaceae* developed by Microgen (Moscow, Russia) (1 × 10^6^ pfu/mL) and administered orally for 10 days [19]. The authors showed that phage lysates could cause inflammatory reactions by, among others, increasing the level of circulating endotoxin as a result of changes in the intestinal microflora and an increase in intestinal permeability. In their research, they showed differentiation in the composition of the microbiome before and 10 days after administration of the phage cocktail. It has been shown to reduce the number of bacteria from *Actinobacteria, Deferribacteres*, and *Proteobacteria* and to reduce bacteria from *Spirochetes*, *Tenericutes* and *TM7*. It is well known that phage administration reduces the number of *Lactobacillus* and *Faecalibacterium* species that are very beneficial to mammalian organisms. The phages used in the studies did not directly affect both of these species, but other phages may lead to microbial changes, which may affect the macroorganism [19].

The virome of the human gut has been studied in a number of diseases with a connection to changes in the composition of known phages from *Caudovirales,* but till now, little is known about any role of the virome in disease [20]. The latest report reveals a highly interactive and dynamic community in the gut microbiome where lytic phages coexist and knockdown targeted bacteria and ultimately modulate the gut microbiome. Moreover, the authors showed that phages have the ability to modulate the bacterial community with influence on the production of metabolites, such as neurotransmitters, amino acids, and bile salts, which are known to affect the mammalian host [33,34]. Norman et al. (2015) showed significant associations between the expansion of the *Caudovirales* phages and specific members of the bacterial community in the IBD disorder [35]. The authors showed that in Crohn’s disease (CD) and ulcerative colitis (UC) patients, the enteric virome demonstrated an abnormal form, and they are disease- and cohort-specific. It did not appear that expansion and diversification of the enteric virome were secondary to changes in bacterial populations. It is believed that changes in the virome may contribute to intestinal inflammation and bacterial dysbiosis and that they take part in inflammatory bowel disease. As the main part of the human virome, the *Caudovirales* phages may play an essential role in the physiology of the intestine and the bacterial microbiome composition. The geographic variation of phages and specific bacteria, as well as unique changes of phages in CD and UC patients (each disease harbored unique phages), were found. An increase in differences in richness and certain taxa of *Caudovirales* phages between CD and UC was observed by the authors. In the CD and UC patients, a significant reduction in bacteria diversity and bacterial richness, as well as the specific virome for CD patients compared to household control, was found. As a consequence of bacterial lysis, via proteins, lipids, nucleic acids pathogen-associated molecular pattern (PAMPs, antigen release and direct interactions between phages and the mammalian host, the chronic intestinal inflammation leading to the destruction of intestinal tissue in IBD pathology have been observed [35].

It additionally has been postulated [36,37,38,39] that phages have unique binding sites to inflammatory mediators. T4 phages revealed that the needle domains contain seven iron ions coordinated by histidine residues. The tail adhesin gp12 mediates adsorption of T4 phages to *E. coli* cells [40], whereas recombinant gp12 binds to LPS and also prevents LPS-induced production of proinflammatory cytokines in mice [41]. It is believed that phages can cross the epithelial cell layer and interact with underlying immune cells. Lehti et al. (2017) described that phages could be internalized by eukaryotic cells by binding to moieties that resemble bacterial phage receptors [42]. The presence of an integrin-binding motif in genetically engineered filamentous phage was shown by Namdee et al. (2018) [43]. Nguyen et al. (2017) showed phage internalization by endocytosis [44] and that they were transferred through the Golgi apparatus [45]. The immunoregulatory effect of T4 phages manifested as a reduction in reactive oxygen species (ROS) production by peripheral blood polymorphonuclear leukocytes stimulated by LPS was demonstrated by Międzybrodzki et al. (2008) [46]. Moreover, it has been shown that NF-kB activity may be modulated by the *Staphylococcus aureus* phage JS25 by inhibition of the production of several proinflammatory cytokines and inhibited NF-kB signaling [47]. Zimecki et al. (2003) demonstrated that the *S. aureus* A20/R phage mediates the costimulatory activity of splenocyte proliferation and proinflammatory cytokine IL-6 production [48]. An in vitro study with *S. aureus* and *Pseudomonas aeruginosa* phages demonstrated production of both pro- and anti-inflammatory cytokines from peripheral blood mononuclear cells following endocytosis of purified phage virions (the prevailing effect being anti-inflammatory) [49]. Additionally, these results are supported by the proposed ability of phage virions to cross the mammalian epithelial barrier in vitro via peptide sequences expressed on the phage capsid surface [3,5,21,44]. Furthermore, the *E. coli* PK1A2 phage can interact with polysialic acid at the surface of the eukaryotic cell lines, which has a structural similarity with polysialic acid polysaccharide of the bacterial host [42]. The *E. coli* PK1A2 phage from the *Podoviridae* family within the supergroup T7, subgroup SP6 is a natural variant of the PK1A phage, which was isolated because of its ability to bind bacteria containing the K1 polysialic acid capsule with a structure identical to the polysialic acid present on mammalian cells. The PK1A2 phage is closely related to the *E. coli* K-1 specific phage K1E and propagated on *E. coli* host strain IH 954 with a highly reduced amount of K1 capsule. The authors showed in in vitro studies that the phages bind to polysialic acid and that they progressively internalize to the live eukaryotic neuroblastoma cell line SK-N-SH, SK-N-AS, human foreskin fibroblast cell line BHK-21 from the American Type Culture Collection (ATTC) [42].

### 3.1. Phages in Crohn’s Disease and Ulcerative Colitis

Fecal and mucosal phage communities have been studied in inflammatory bowel disease (IBD), a chronic disorder of the gastrointestinal tract [20]. The composition of gut bacteria and their interaction with the host immune system is believed to be central to its pathology, but the etiology of IBD is still poorly understood. Two types of IBD are UC and CD. It is believed that disease-specific patterns of the order *Caudovirales* are linked to Crohn’s disease and ulcerative colitis and that phages belonging to this order play a central role in shaping the microbiome in inflammatory bowel disease [20]. Lawrence et al. (2019) revealed that there is an increase of phages in IBD patients compared to their healthy controls with differences in such populations between UC and CD patients [50]. Manrique et al. (2017) conclude that a proper balance between lytic phages and activated prophages is essential to protect against IBD development [29]. Further, it was postulated that the development of *Caudovirales* phages in the rectal mucosa of UC was correlated with intestinal inflammation [28]. Rectal mucosa of 91 patients with UC and 76 healthy controls was examined. The *Escherichia* phages and *Enterobacteria* phages were more abundant in mucosa UC compared to healthy controls. Researchers suggested that the expansion of mucosal phages may have an essential role in UC pathogenesis. The most important finding of Zuo et al. (2019) was the detection of the abundance of *Caudovirales* phages in gut mucosa UC patients in comparison to healthy controls, whereas a decrease of evenness, diversity and richness was observed within *Caudovirales,* and there was a clear indication of dysbiosis in mucosal virome in UC patients [28]. Gogokhia et al. (2019) have shown that phages isolated from a human patient with IBD stimulated an immune response in the mouse gut (induction of proinflammatory genes, expansion of CD4+ and CD8+ T cell population in gut lymph nodes) [18]. A broad range of *Caudovirales* phages stimulated IFN-gamma production in vitro. The authors suggest that *Caudovirales* phages can function as adjuvants. In addition, they showed that patients with IBD who do not respond to fecal microbiota transplantation (FMT) have higher levels of *Caudovirales* phages. Interestingly, the same phages in a mouse model of colorectal cancer suppressed tumor growth [18,51].

### 3.2. Gulf War Illness (GWI) and Phages

GWI is associated with inflammatory bowel disease and other poorly characterized medical conditions. Using a murine model of GWI, Seth et al. showed alterations in enteric viral populations, especially phages, in that syndrome [52]. GWI mice had a decreased abundance of the *Microviridae* phage with an increased relative abundance of the *Siphoviridae* and *Myoviridae* phage in comparison to control mice. Interestingly, virome abnormalities correlated with elevated proinflammatory cytokine levels (IL-6, IFN-gamma and IL-1beta) and activated TLR signaling. Antiviral (but not antibiotic) treatment partially corrected those abnormalities while both treatments restored gut epithelial membrane integrity [52].

### 3.3. Fecal Microbiota Transplantation

Fecal microbiota transplantation (FMT) offers the possibility to restore healthy gut function through a fecal slurry from a healthy individual to the colon, cecum or duodenum of the recipients in the treatment of recurrent *C. difficile* infection (CDI) [53], with success rates of 80–90% [54] when the CDI symptoms were eliminated for up to 6 months [55]. Interestingly, phage transfer in FMT in *C. difficile* infection brought beneficial results in the reduction of disease symptoms [55,56]. A nasojejunal tube inserted by gastroscopy, using a fiberoptic system (Olympus, Germany) and controlled by an APPLX smart/vision system (Fresenius KABI, Germany), in 5 patients with chronic CDI was used [55]. A single dose of fecal filtrate (FFT) was administered at 30 min after the 1000 mL of Klean-Prep intestinal lavage solution (Norgine, Germany). The fecal samples from 5 patients and 5 donors were tested for the microbiome, virome and proteome profiles. Fecal samples were taken from the patient before and at 1 week and 6 weeks after FFT. A restored normal stool microbiome and CDI symptom elimination, including diarrhea, as well as longitudinal changes in the microbial and viral community in all 5 patients after FFT was observed [55]. Furthermore, Kao et al. (2019) supported the observations of Ott et al. (2017) on symptom elimination in CDI patients [57]. Kao et al. (2019) presented the randomized, double-blind pilot study on the effect of lyophilized sterile fecal filtrate (LSSF) vs. lyophilized fecal microbiota transplantation (LFMT) on recurrent *C. difficile* infection (RCDI). This single-center pilot study aimed to determine if the FMT live microbes are necessary for clinical efficacy. The age > 18 years; last 3 episodes of RCDI; positive for *C. difficile* toxin; and recurring diarrhea after symptom resolution, finally following 10 days of anti-CDI therapy and CDI infection under symptomatic control with <3 loose/unformed stools/24 H was the main inclusion criteria of the rCDI infection. On the other hand, the main exclusion criteria were taken into account: fulminant CDI; chronic diarrheal illness, dysphagia; ileus or bowel obstruction; pregnancy; active infection requiring antibiotic therapy, and <6-month life expectancy. Two groups of 5 patients were qualified for therapy under direct observation in the clinic. A single dose of 15 capsules of LFMT or LSFF, according to in-group assigned, was administered. All patients were seen in the clinic at week 0 for treatment and 1, 4, 8 and 24 weeks after treatment. The *C. difficile* testing was provided for evaluating the recurrence of diarrhea (≥3 unformed bowel movements/24 h). The 75% (¾) patients of the LSFF and 80% (4/5) patients of the LFMT group was demonstrated the primary outcome of the therapy. The authors pointed out that this preliminary study shows that the clinical efficacy of FMT in rCDI therapy did not depend on the presence of live microbes in the donor stool [57].

Zuo et al. (2018) demonstrated the results of FMT in 24 patients with CDI and 20 healthy donors [56]. The metagenomic sequencing of VLPs and 16Sr DNA sequencing was used to determine enteric virome and phage-bacteria interaction. The Spearman correlations for *Caudovirales* bacterial taxa were used; phages from this order were the most abundant in both CDI patients and healthy donors. CDI patients demonstrated a significantly higher abundance of *Caudovirales* at the species level, as well as a decrease in their richness and evenness compared to healthy donors. On the other hand, the decreased abundance and diversity of *Microviridae* but increased abundance of *Anelloviridae* compared with donors was observed before FMT treatment. A decrease of *Caudovirales* abundance and an increase in *Microviridae* in nine CDI patients with profound differences in virome composition among them was observed after FMT administration. The authors studied the effect of donor *Caudovirales* richness on treatment response, and they found that the CDI patients achieved a response to FMT when their *Caudovirales* richness was lower than in the donor. The increased frequency of bacterial families, such as *Lachnospiraceae* and *Ruminococcaceae*, in the stool of FMT CDI patients, was found. In the CDI patients after FMT, the shift of the microbe community from low to high bacterial richness and diversity and from low to high *Caudovirales* diversity was shown. A more positive correlation between *Caudovirales* species, such as *Burkholderia, Plantothrix, Pseudomonas, Moraxella* and *Halomonas* phages, with bacterial families of *Proteobacteria* and *Actinobacteria* in CDI patients, was found. The authors summarized that after FMT, the treatment response in CDI patients was associated with a high colonization level of donor-derived *Caudovirales* [56].

Other researchers also investigated the correlation between the outcome of FMT and the abundance of phages in patients with ulcerative colitis [18]. As mentioned, patients with clinical success of FMT indicated a lower abundance of the *Caudovirales* phage [18].

### 3.4. Phages in Diabetes

The role of gut phages in type 1 diabetes (T1D) has recently been explored [22,25]. The group of patients was 10 children who exhibited autoantibodies (six seroconverters with no progression to T1D and four children who developed T1D). The control group was eight non-seroconverted HLA-matched individuals [25]. The 63 *E. coli* phage species, most temperate, were detected in T1D cases and a control group. Only in a few samples were the virulent *Enterobacteria* phage IME10 and *Enterobacteria* phage 9 g [25]. In a study of HLA-matched infants up to 3 years of age, nearly all intestinal *E. coli* phages were temperate and only in a few samples were lytic *Enterobacteria* phages found [25]. Release of amyloid from intestinal *E. coli* bacteria due to prophage activation was correlated with the development of T1D-associated autoimmunity, suggesting a role of phages in the modulation of immunity [22,25]. The *E. coli* MG-1655 strain as host for prophage induction as well as temperate *E. coli* phage lambda from their own collection was used [25]. The influence of prophage on amyloid release in *E. coli* biofilm induced by mitomycin C in in vitro study with the amyloid-diagnostic dye Congo red (CR) was investigated. A significant increase in CR absorbance on binding to biofilm supernatant after prophage induction as compared with control samples was observed. The authors postulated the importance of potential diabetogenic *E. coli* prophages in the autoimmunity and T1D progression by the pronounced amyloid release from microbial biofilm in vitro and suggested that the same phenomenon may occur in children with T1D [25]. Due to the predominance of temperate phages in the gut, this finding practically does not apply to phage therapy.

The first study of the correlation of gut phages with type 2 diabetes (T2D) indicated an increase in the number of gut phages in T2D and identified 7 phage operational taxonomic units (pOTUs) specific to T2D [58]. The bacterial hosts of pOTUs were *Enterobacteria, Escherichia, Lactobacillus, Pseudomonas* and *Staphylococcus*. Then, 116 T2D samples from Chinese adult individuals were analyzed, and all 7 pOTUs were correlated with T2D [58]. Additionally, an increase in the abundance of *Escherichia, Clostridium, Lactobacillus, Pseudomonas* and *Staphylococcus* phages (temperate and lytic) in the human gut with type 2 diabetes was detected [22]. However, the authors did not discuss how drugs received by patients with diabetes may affect gut phages. In particular, treatment regimens, including metformin, can confound the correlation between type 2 diabetes and the microbiome [59], while this drug has also been shown to have antimicrobial effects [60]. Further studies are necessary to explain the correlation between gut phageome and T1D and T2D risk. Interestingly, a pilot study in mice showed that a similar fecal filtrate as in Ott et al. 2017 [55] decreased the symptoms of metabolic syndrome in the mice—which was proposed to be associated with phage-mediated gut microbiota manipulation [61]. The authors present the results on the role of fecal virome transplantation (FVT) of the type 2 diabetes symptoms and obesity in the murine model. The male C57BL/6 N Tac mice were divided into five groups (with a low-fat diet (LF) as a control, high-fat (HF) diet, HF+ ampicillin (Amp), HF+ Amp+ FVT, and HF+ FVT) were used in the study. The HF + FVT and HF + Amp + FVT mice were treated with FVT by oral gavage at weeks 6 and 7 of a study. Ampicillin was administered 24 h before the first FVT treatment. A significant decrease in weight gain compared to the HF group after the first FVT at 6 weeks in the HF + FVT group and significant gut microbiota (GM) composition shifts were observed. The authors showed the weight gain reduction and normalization of blood glucose parameters in mice with an obese phenotype after cecal virus communities transfer as a result of GM changes of FVT administration [61].

### 3.5. Phages in Other Pathologies

Some authors support the concept of phages as human pathogens because of their interactions with eukaryotic cells and proteins, which can lead to human diseases. Phages against *E. coli*, *Staphylococcus, Klebsiella, Bacillus* and *Paenibacillus* were detected in animal blood after oral administration. In human biological fluids, the *Staphylococcus* phage StB2 and *Shigella* phage SfIV were detected by the authors in the cerebrospinal fluid (CSF) in patients with neurodegenerative pathologies such as multiple sclerosis [62]. Available data on phage penetration to different organs, i.e., the brain, allows a connection to be made to phages circulating in human biological fluids in neurodegenerative diseases [62]. The authors speculate that the prion domains present in phages may be involved in interactions with eukaryote proteins and in protein misfolding in humans [63] and their connection with autoimmune and neurodegenerative disorders, such as Alzheimer’s disease (AD), Parkinson’s disease (PD) and amyotrophic lateral sclerosis [62]. The authors identify prion-like domains in a variety of phages associated with the human microbiota by using a computational algorithm, prion-like amino acid composition, and identify 5040 putative prion protein sequences [63]. The most abundant prion-like domains were found in all phages from the *Caudovirales* order, but most frequently in the *Podoviridae* family. Five groups of phage proteins that are involved in interactions with bacterial cells, such as attachment and proliferation, DNA replication and protein assembly, as well as the release of prion-like domains, were analyzed.

The presence of prion-like domains in the phage proteins of head, neck, sheath, baseplate, as well as in phage-encoded endolysins in the *Siphoviridae* family was identified by the authors [63]. Phage M13 was found to possess multiple prion-like domains within the attachment protein G3P. It was shown that the abundance of lytic *Lactococcus* phages was higher in patients with PD than in healthy individuals. In the indirect interaction with the host microorganism, phages can affect the human host through induction of increased intestinal permeability by reduced *Lactobacillus* spp. and *Faecalibacterium* spp., which are important regulators of the intestinal barrier and create the possibility for phage circulation in the CSF. The possible influence of phages on PAMPs such a LPS, peptidoglycan and bacterial amyloid into the bloodstream during oral phage administration by an increased level of plasma cell-free bacterial DNA has been pointed out [62]. The authors mention that phage prion-like proteins are involved in the interaction between phages and bacterial cells and postulate the possible association of prion-like domains with human diseases, as well as the necessity of further experimental studies [62,63].

Ghose et al. (2019) revealed the presence of virome of CSF from 20 individuals, including 17 without infection and 3 with a central nervous system (CNS) infection and compared it with viromes found at different body sites [64]. To determine the counts of VLPs in the tested specimens, the procedure elaborated for virus isolation from environmental samples [65] was modified. The VLPs particles of 10^4^ per mL of CSF were identified in the cohort by epifluorescence microscopy compared with nonsterile saliva samples from another cohort, which contained 10^6^ VLPs per mL [64]. The CSF viromes were analyzed with the largest available dataset (IMG/VRv2.0); 68.3±0.7% homology was found. The majority of the CSF phages were classified as the order *Caudovirales* and *Myoviridae, Siphoviridae* and *Podoviridae* families. The authors found that the virus-like particles were present in the CSF of all 20 studied subjects. Only 2 virus assemblies as *Myovirus* and *Siphovirus,* were present in all 20 subjects. One of the two viruses that were assembled from all 20 subjects includes a putative 66.4 kb *Myovirus* that has restriction/modification enzymes and includes an integrase suggesting it has a lysogenic lifestyle. The other includes a putative 37.0 kb *Siphovirus* that has a transposase that also suggests it has a lysogenic lifestyle. It has been shown that all observed virus families were present in the different specimen types and that the body surfaces can be grouped into two separate clusters, one containing urine, feces and saliva, and the other including CSF, plasma, body fluids and breast milk. Low-virome alpha diversity was detected in CSF, plasma, body fluids, and breast milk and high virome alpha diversity were detected in urine, feces and saliva of examined individuals. Infection status does not specifically define CSF viral ecology. There were no trends in beta diversity in the viromes between CSF specimens from individuals without and with infection [64].

In other disorders, such as malnutrition, alteration of the gut virome was reported reflecting a reduced diversity of viromes [31]. Burgener et al. (2019) postulated that the presence of filamentous Pf phages produced by *P. aeruginosa* strains might be associated with chronic infection and increased antibiotic resistance in cystic fibrosis (CF) patients [66]. The presence of Pf phages with an average of 4,4x 10^9^ pfu/mL sputum was detected in the sputum of 37% of older patients with chronic *P. aeruginosa* infection and advanced lung disease in comparison with patients without Pf phage presence. Sputum samples from two well-characterized groups of CF patients have been studied for the presence of Pf phages. It has been shown that 26.5% (n = 9) of patients from 34 CF patients from a Danish population were Pf phage-positive compared to 36.2% (n = 21) being Pf phage-positive from 58 Stanford CF patients. The authors show that *Pseudomonas* strains collected from patients that were Pf phage-positive exhibit significantly increased resistance to amikacin, aztreonam and meropenem, which are commonly used in *P. aeruginosa* infection in CF patients. The overall FEV_1_ values (forced expiratory volume), BMI (body mass index) and other metrics of CF-disease were not different in Pf phage-positive and Pf phage-negative patients. The authors report that higher levels of Pf phages are associated with increased patient age, chronic *P. aeruginosa* infection, lower FEV_1_, and an increased antibiotic resistance profile in a group of cystic fibrosis patients [66]. Further fascinating data in terms of temperate *P. aeruginosa* Pf phages was provided by Sweere et al. (2019) [67]. It turns out that phages may be involved in an unsuspected, directly pathogenic function through promoting bacterial infections. Endocytosis of the temperate *P. aeruginosa* phage (Pf) by dendritic cells and other leukocytes in human and murine models triggers viral pattern recognition receptors (PRRs), which suppress bacterial clearance. Endocytosis of Pf phages both in vitro and in vivo resulted in the production of phage RNA, which, in turn, triggered Toll-like receptor 3 (TLR3) and TIR-domain-containing adapter-inducing interferon-β (TRIF)-dependent viral PRRs, driving type I interferon production, inhibiting tumor necrosis factor (TNF) production, and suppressing phagocytosis. These effects result in more frequent infection and may have broad relevance beyond *P. aeruginosa* wound infections. Pf phages are also abundant in *P. aeruginosa*-associated respiratory infections and are likely to be present in other *P. aeruginosa* infections [67]. However, to date, lytic phages have not been reported to suppress phagocytosis or local inflammatory cytokine secretion.

In other studies, the respiratory virome in 63 patients with chronic obstructive pulmonary disease (COPD) exacerbations was investigated by metagenomic next-generation sequencing (mNGS) and qPCR [68]. The most prevalent phyla in the respiratory bacteriome were: *Proteobacteria, Firmicutes, Actinobacteria* and *Bacteroidetes.* The incidence of viral pathogens was 26%. Patients with viral pathogens had reduced percentages of phages compared to patients without viral pathogens, with 0% and 79% phages, respectively. The studies suggested that lower phage abundance may be due to viral expansion. Additionally, virome dysbiosis may be accompanied by bacteriome dysbiosis. No correlation between phage abundance and COPD exacerbations of a viral cause was observed [68].

The major findings from studies of phages in the human body in different diseases are summarized in Table 1.

### 3.6. The Abundance of crAssphage and Human Health

Interestingly, recent data did not show a correlation between the abundance of crAssphage in the gut microbiome and human health and human diseases [17,23]. The abundance of crAssphage in the gut was estimated in the LifeLines-DEEP samples of 1135 individuals from the north of the Netherlands [23]. The studies examined the correlation between the abundance of crAssphage and 207 human variables (78 dietary factors, 41 intrinsic factors, 39 diseases, 44 drug groups, 5 smoking categories) and 490 microbial taxa. The study indicated the most correlation of crAssphage with microbial taxa included the family *Prevotellaceae*. This is compatible with the previous suggestion that this phage infects the *Bacteroidetes* phylum. A weak correlation between crAssphage and several diet categories, including protein, carbohydrates and caloric intake, was observed. The LifeLines-Deep study did not find a significant relationship between crAssphage and any health or disease parameters, so this phage may be a part of the normal human gut virome. Additional analysis of fecal samples from 45 healthy individuals from 4 cities in two continents reveal crAssphage-positive results in half of the individuals suggesting the prevalence of this phage’s population in the world [23]. Other results are compatible with the study of Edwards et al. (2019), which showed no significant differences in the crAssphage-positive ratio (11.5% vs. 8.3%) and viral loads (Ct value: 29.7 ± 0.5 vs. 29.9 ± 0.6) in fecal samples between diarrhea and healthy adults. The study suggests no association between crAssphage in the human gut and gastrointestinal disease [17].

## 4. Impact of Phage Intake on Gastrointestinal Human Health

Febvre et al. (2019) revealed that supplemental phage intake had no significant impact on gut microbiota and overall health status [69]. In a double-blinded placebo-controlled study, they applied a phage cocktail *E. coli* for 28 days to healthy individuals. They determined the effect of a phage cocktail on gut microbiota and markers of intestinal and systemic inflammation. The applied phage did not globally disrupt the microbiota. However, during the study, specific bacteria were altered; that is, there was an increase in the members of *Eubacterium* and a decrease in the taxa related to *Clostridium perfringens*. Moreover, inflammatory markers and lipid metabolism were unaltered. A small decrease in circulating IL-4 was observed [69].

In a small intestinal in vitro model, researchers showed similar results [70] as Febvre et al. [69] of using strain-specific coliphages to target *E. coli*. The phage cocktail *E. coli* was similar to ciprofloxacin in reducing *E. coli* by 2–3 log but had a much milder influence on commensal non-targeted bacteria compared to the antibiotic [70]. Considering that only *E. coli* phages were applied in both studies [69,70], one could question whether it really is surprising that no significant global phenotypic effects are observed since the commensal *E. coli* only represents a minor fraction of the normal gut microbiota [71]. Both Hsu et al. (2019) [33] and Fazzino et al. (2020) [72] have shown how other than coliphages indirectly may affect other members of the gut microbiota. Hsu et al. (2019) indicated in a mouse model that lytic phages not only knockdown their bacterial hosts but also affect non-susceptible commensal bacteria in the gut through cascading effects. Fazzino et al. (2019) demonstrated in laboratory experiments that the attack of *Salmonella enterica* with specific P22 vir phage delayed community growth with little effect on final species ratios. On the other hand, the *E. coli*-specific T7 phage attack altered the final species ratios in favor of *S. enterica* and caused a small delay of community growth.

The PHAGE study, a double-blinded placebo-controlled study, defined the safety and tolerability of phages in healthy adults with mild to moderate gastrointestinal distress [73]. Clinic visits of participants took place at the Colorado State University Human Performance Clinical Research Laboratory before and after each 28-day treatment. Participants used an *E. coli* phage cocktail or placebo (1 capsule daily) for 28 days, followed by a 2-week washout period and 28 days of the opposing treatment. The study showed the safety and tolerance of phages in the human population being studied and even some anti-allergic effects (lowering of cytokine IL-4 serum levels) [73]. The study suggests that phages may be applied as a dietary supplement in healthy individuals with mild to moderate gastrointestinal distress without causing exacerbation of symptoms. Future analyses may explain the effect of phage consumption on the gut microbiota and intestinal and systemic inflammatory markers [73].

## 5. Conclusions

Recent data demonstrating the presence of phages in the human body open new and exciting perspectives for research on the actual significance of such “body phageome” [5,74]. Currently, while the protective effects of such phages have been initially suggested [75,76], the recent reports mentioned above also suggest potential pathogenic effects of such phages. Moreover, there is also ample evidence indicating that phages do not adversely affect the immune system and do not cause any harmful effects, even those present in cerebrospinal fluid [64]. We have pointed out that the effects of phages on the immune system may be phage-specific [77], which is probably most close to the truth. Further studies are necessary to shed more light on those phenomena, which would determine the multifaceted role of endogenous phages and their significance for further progress of phage therapy.

## Figures and Tables

**Table 1:** Studies of phages in the human body in different diseases.

Disease	The Most Important Finding	Reference
Inflammatory bowel disease (IBD)	Expansion of *Caudovirales* phages in enteric virome of IBD patients	[35]
Ulcerative colitis (UC)	The detection of the abundance of *Caudovirales* phages in gut mucosa UC patients, whereas a decrease of evenness, diversity and richness of *Caudovirales* phages and an indication of dysbiosis in mucosal virome in UC patients	[28]
Gulf war illness (GWI)	GWI mice had decreased abundance of the *Microviridae* phage with increased abundance of the *Siphoviridae* and *Myoviridae* phage in the enteric viral population	[52]
Type 1 diabetes (T1D)	Predominance of temperate phages in the gut of children. The importance of diabetogenic *E. coli* prophages in the autoimmunity and T1D progression	[25]
Type 2 diabetes (T2D)	Increase in the number of gut phages in T2D adult individuals. Phages specific to *Enterobacteria, Escherichia, Lactobacillus, Pseudomonas* and *Staphylococcus,* were detected in T2D patients. T2D-related factors in the gut of T2D patients cause temperate phages to switch to the lytic cycle	[58]
Autoimmune and neurodegenerative disorders	Phages circulate in human biological fluids in neurodegenerative diseases. *Staphylococcus* phage and *Shigella* phage were detected in cerebrospinal fluid in neurodegenerative disorders. Prion domains present in phages may be involved in interactions with eukaryote proteins and in protein misfolding in humans and their connection with autoimmune and neurodegenerative disorders	[62]
Central nervous system infection	There were no trends in diversity in the viromes between cerebrospinal fluid specimens from individuals without and with central nervous system infections. The majority of phages of the cerebrospinal fluid were *Caudovirales.* Temperate *Myovirus* and *Siphovirus* phages were present in individuals without and with infection	[64]
Malnutrition	Reducing the diversity of gut viromes in children	[31]
Cystic fibrosis (CF)	The presence of filamentous *Pseudomonas* Pf phages in sputum may be associated with chronic infection and increased antibiotic resistance in CF patients	[66]
